# Can smoking cause impotence? a radiological retrospective cohort study comparing internal pudendal artery calcification on CT in male smokers versus non-smokers

**DOI:** 10.1007/s11845-022-02948-5

**Published:** 2022-02-17

**Authors:** Emma Tong, Caoimhe McDonnell, Kate Hunter, Kevin Sheahan, William C. Torreggiani

**Affiliations:** 1 Department of Radiology, Tallaght University Hospital, Dublin, Ireland; 2 Department of Radiology, St. James Hospital, Dublin, Ireland; 3 Department of Radiology, Beaumont Hospital, Dublin, Ireland

**Keywords:** Calcification, Erectile dysfunction, Smoking, Vascular

## Abstract

**Background and aims:**

This retrospective cohort study evaluated the degree of pelvic inflow and internal pudendal artery (IPA) calcification in male smokers versus non-smokers. As erectile dysfunction (ED) is strongly associated with IPA vascular, we wanted to investigate radiologically if there was a statistically significant difference in the degree of IPA calcification in smokers and potentially be a contributing factor in the cause of ED.

**Methods:**

CT studies of 100 men aged between 40 and 60 years of age were blindly reviewed and assigned a calcium score of their vascular calcification levels. We compared scores of 50 smokers versus 50 non-smokers. The Mann Whitney *U* test statistic was used to test for a statistical difference in calcification score between the smoking and non-smoking groups.

**Results:**

Results show a statistically significant association between smoking and pelvic inflow and IPA calcification. The Mann Whitney *U* test demonstrated a statistically significant higher calcium score in the smoking group (mean = 4.8, SD 3.7), versus the non-smoking group, (mean = 1.8, SD 1.9) (*U* = 701.5, *p* < 0.05).

**Conclusions:**

This research is the first of its kind based on an extensive literature review. The association between vascular calcification and smoking is well established, in addition to the direct relationship of IPA calcification and ED. This unique study has demonstrated an increased rate of IPA calcification in smokers with a potential inferred association with ED. Findings represent a novel and useful deterrent for health authorities to include in anti-smoking campaigns.

## Introduction

In this paper, we aimed to examine whether there was a clear cause and effect between smoking and pelvic inflow internal pudendal artery (IPA) calcification, and to demonstrate whether this link is both radiologically apparent and statistically significant. As erectile dysfunction (ED) is strongly associated with IPA calcification, we further hypothesised if we could demonstrate radiological evidence between smoking and ED [1].

### Erectile dysfunction

Erectile dysfunction is an increasingly common condition, whereby erection cannot be induced or maintained sufficiently. It is primarily a vascular condition but also has psychological, neurologic, and hormonal causes. ED has an overall prevalence in men of approximately 16%, and becomes more prevalent with age — rising from 8% between ages 20 and 30 years to 37% between ages 70 and 75 [2].

ED is associated with many of the same risk factors as for coronary artery disease, chiefly smoking and obesity, but also diabetes, dyslipidaemia, and chronic kidney disease [1]. Treatment of ED is largely focused on the underlying aetiology; therefore, successful identification of the cause is important in its management. Given that lifestyle factors such as smoking, obesity, and hypertension are important risk factors, cessation and management of these are essential for effective management and prevention of ED [3].

### Vascular calcification

Vascular calcification (VC) is an important cause of morbidity and mortality and is commonly associated with ED [2]. Chiefly, it is associated with atherosclerosis, as well as chronic kidney disease and diabetes mellitus, which all contribute toward VC development. VC incidence and severity increase with age and is more common with osteoporosis, obesity, smoking, and lack of exercise [4].

VC can be assessed radiologically in several ways, while being both non-invasive and diagnostically accurate. Multi-slice CT is very sensitive for detection of VC and also allows for accurate and precise quantification [5]. Coronary artery disease (CAD) calcification is an area which has been well studied and received substantial investigation, as identification can allow for significant reduction in atherosclerotic CVD (ASCVD) risk. CAD is best assessed using cardiac CT, which is now very common practice [6].

VC in peripheral arterial disease (PAD) is also routinely assessed radiologically utilising CT angiography (CTA), although other modalities can be used, such as magnetic resonance angiography (MRA) and Doppler ultrasound [7].

Identification and quantification of calcification and stenosis in the internal pudendal artery (IPA) however have not been well studied in comparison according to our extensive literature review. Digital subtraction angiography (DSA) assesses stenosis and variation in vascular anatomy and is required for penile revascularisation candidates. IPA atherosclerotic lesions have also been used to predict ED onset [8].

### Smoking

Cigarette smoking remains a global health epidemic with associated detrimental effects, and is one of the leading causes of premature mortality in the world, contributing to around 18% of all deaths. It imposes enormous economic costs to society, both directly from health-care needs and indirectly from loss of productivity. While tobacco use represents an important public health issue worldwide, it is particularly important in the WHO European Region, where there is the highest proportion of tobacco use in the world, with an estimated 209 million people (or 29%) currently smoking [9].

The WHO estimate that 12% of all deaths among adults ages > 30 years and above can be solely, or partly attributable to tobacco use, and figures for men were more than double compared with women, (16% and 7% respectively) [10].

Many European countries are trying to reduce the economic and health costs of this major epidemic but the majority of countries still face a challenge in designing and implementing comprehensive and sustainable tobacco-control strategies. Member states of the WHO European Region have implemented multiple policy frameworks to reduce smoking rates, such as indoor smoking bans, increased resources to support smoking cessation, and limiting tobacco marketing; however, the levels achieved so far have been insufficient to reach the target prevalence reduction of 30% by 2025 [11].

Tobacco use significantly increases the rate and burden of multi-system vascular calcification. One of the most well studied areas pertains specifically to coronary artery calcification [12]. There are also many studies examining vascular calcification within the intra-abdominal vessels [13] and intracranial vessels [14]. However, radiological assessment of internal pudendal arterial calcification (IPA) is not very well researched. Also, there is a dearth of literature available assessing the potential link between smoking, IPA calcification, and subsequent erectile dysfunction. Perhaps if there was a clear scientific link between the risk of smoking and erectile dysfunction, health authorities such as the WHO could use this evidence as a novel deterrent in anti-smoking campaigns to further help reduce smoking rates in Europe, especially given that young men are the most prevalent group to smoke, and die of smoking related diseases in the European Region [9].

## Materials and methods

A manual search was performed on our picture archiving and communication system (PACS), of all male patients, aged between 40 and 60 years of age at the time of imaging, who underwent computed tomography (CT) examination of the abdomen and pelvis, from January 2019 to April 2020 inclusive. We collected patient data from 100 men, which included 50 smokers and 50 non-smokers. Non-smokers were defined as those who strictly never smoked. Data was obtained from the patient’s electronic patient record. The data included medical record number, date of birth, smoking status, date of CT scan, renal impairment status, and whether the patient was a diabetic. Patients with any form of renal impairment, or who were diabetic were excluded from the study. Any ex-smokers, or smokers who smoked “vapes,” were also excluded from the study sample. The final data was then stored in a password protected excel file, then anonymised and randomised.

The 100 CT studies were then blindly reviewed by a consultant radiologist and assigned a calcium score, ranging from 0 to 10, 0 representing no arterial vascular calcification and 10 representing maximal calcification. Patient demographics were blinded to the radiologist, specifically age and smoking status was not known at the time of vascular calcification scoring.

A 10-point scale was devised to classify the degree of calcification. A patient was awarded 10 points when calcification involved all pre-specified vessels, and 0 represented no calcification of any pre-specified vessel.

The patients were assigned 1 point for any arterial vascular calcification in each of the following vessels:

- abdominal aorta upper (defined as superior to the level of the renal arteries) — 1 point.
- abdominal aorta lower (defined as inferior to the level of the renal arteries to the iliac bifurcation) — 1 point.
- left and right common iliac — 1 point each for the left and right, totalling to 2 points.
- left and right external iliac — 1 point each for the left and right, totalling to 2 points.
- left and right internal iliac — 1 point each for the left and right, totalling to 2 points.
- left and right internal pudendal arteries — 1 point each for the left and right, totalling to 2 points.

The scores of the 50 smokers and 50 non-smokers were then analysed and compared. The Mann Whitney *U* test statistic was used to test for a statistical difference in calcification score between the smoking and non-smoking groups using Microsoft Excel Software.

## Results

We collected data from 50 male smokers, and 50 male non-smokers. The age range was 43–60 years of age in the smoking group; the median age was 54 years (SD 5.6 years). The age range was 40–60 years of age in the non-smoking group; the median age was 51 years (SD 5.9 years). None of the patients had any history of diabetes. And all 100 patients had an eGFR of > 90, indicating no significant renal impairment. All patients underwent CT scanning of the abdomen and pelvis during 2019 or 2020 using conventional CT techniques.

The range of calcification scores in the non-smoking group was 0–8, with a single outlier scoring 8. Excluding this outlier, the range was 0–5, with a mean of 1.8 (SD 1.9). A total of 17/50 patients (34%) in the non-smoking group scored a 0/10 for vascular calcification. Only 6/50 patients (12%) in the non-smoking group demonstrated IPA calcification (either unilateral or bilateral).

The range of calcification scores in the smoking group was 0–10, with a mean of 4.8 (SD 3.7). Only 11/50 patients (22%) in the smoking group scored a 10/10 for vascular calcification. In addition, 25/50 patients (50%) in the smoking group demonstrated IPA calcification (either unilateral or bilateral).

**Fig. 1:**
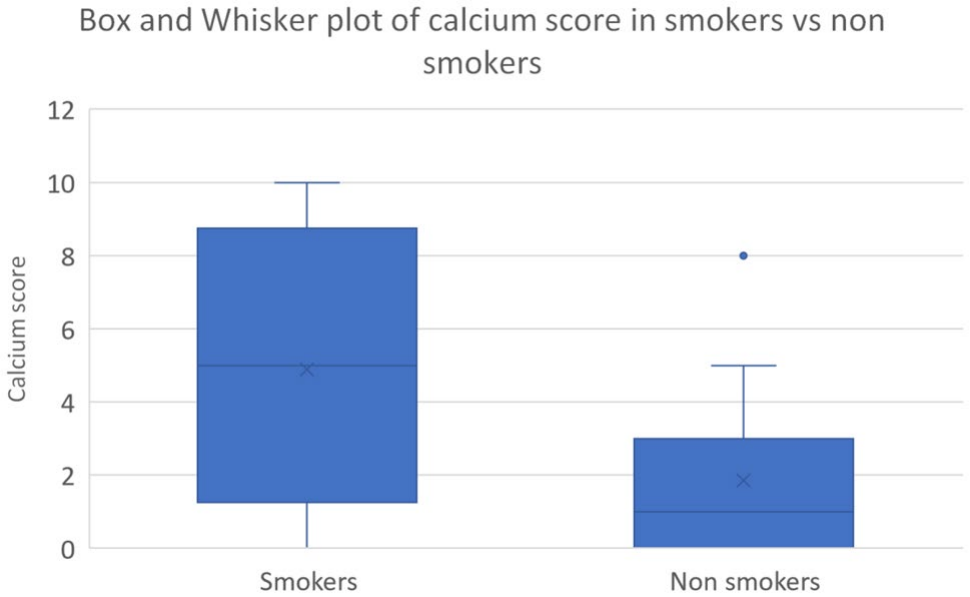
Box and whisker plot of calcium score in smokers vs non-smokers

Figure 1 illustrates the wider range and higher calcium score in the smokers group, ranging from 0 to 10, in comparison to non-smokers calcium scores ranging from 0 to 5 (apart from a single outlier scoring 8). The median calcium score for smokers was higher at 5, and lower for non-smokers at 1.

**Fig. 2:**
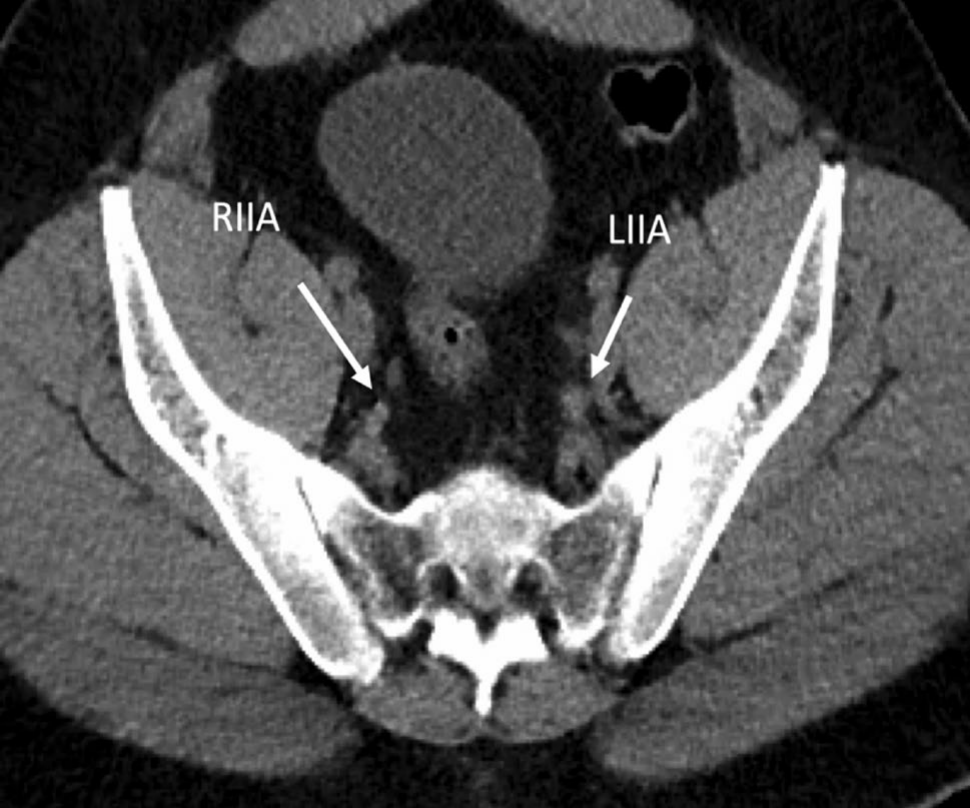
Non-contrast CT images of a 41-year-old male non-smoker, who scored 0/10 on calcium scoring. No arterial vascular calcification was demonstrated. RIIA/LIIA right internal iliac artery/left internal iliac artery

**Fig. 3:**
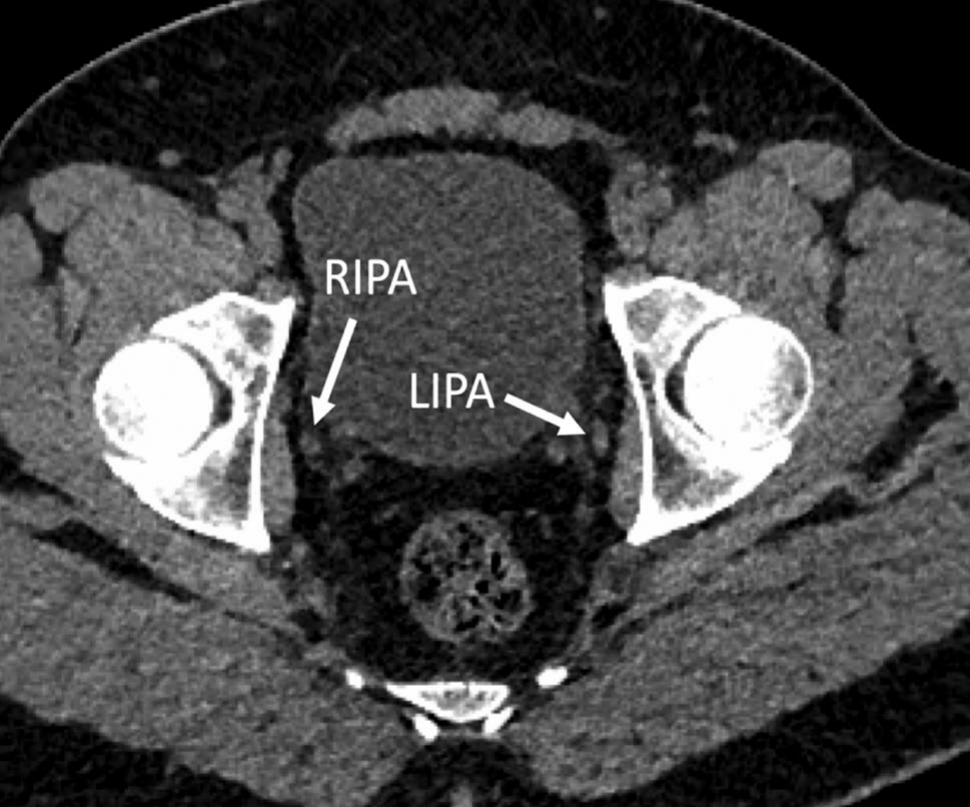
Non-contrast CT images of a 41-year-old male non-smoker, who scored 0/10 on calcium scoring. No arterial vascular calcification was demonstrated. RIPA/LIPA right internal pudendal artery/left internal pudendal artery

The results from this study showed a statistically significant association between smoking and arterial vascular calcification throughout the abdomen and pelvis, and specifically within the internal pudendal artery (Fig. 2). The Mann Whitney *U* test demonstrated a significantly higher calcium score in the smoking group (mean = 4.8, SD 3.7), versus the non-smoking group, (mean = 1.8, SD 1.9) (*U* = 701.5, *p* < 0.05) (Fig. 3).

## Discussion

**Fig. 4:**
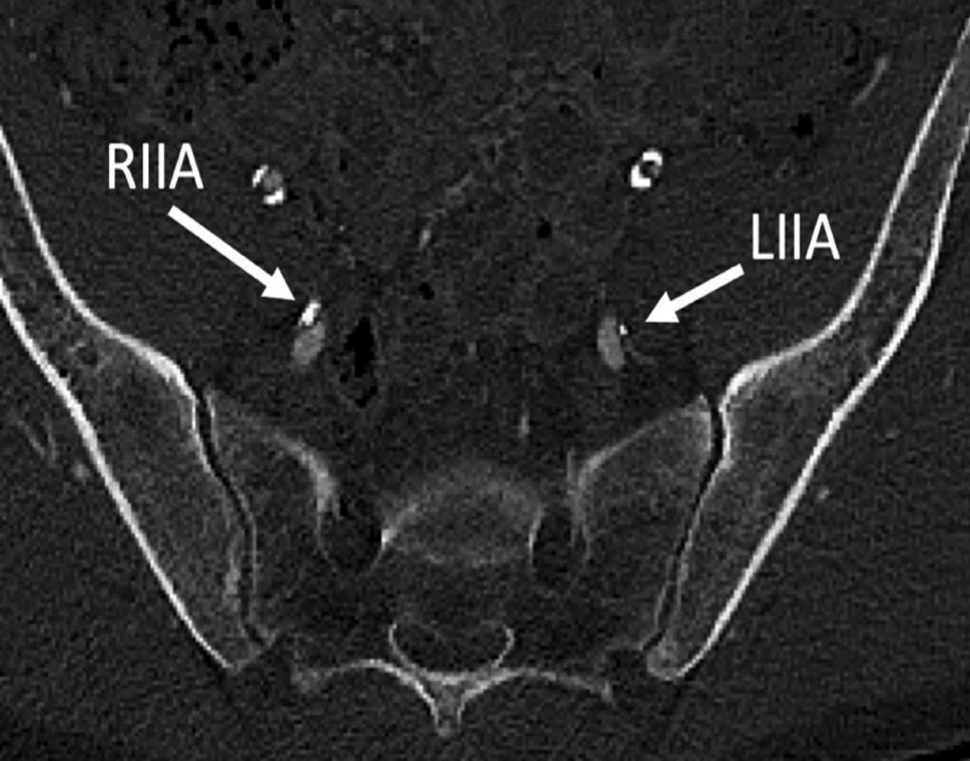
Post-contrast CT images of a 52-year-old male smoker, who scored 10/10 on calcium scoring. Heavy arterial vascular calcification was demonstrated within multiple vessels bilaterally, including the proximal and distal abdominal aorta, common iliac arteries, internal and external iliac arteries, and the internal pudendal arteries. RIIA/LIIA right internal iliac artery/left internal iliac artery

This research has shown a clear and statistically significant increase in pelvic inflow vascular calcification, and specifically of the IPA in male smokers, aged between 40 and 60, as compared to their non-smoking counterparts. This research is also the first of its kind based on an extensive literature review (Fig. 4).

**Fig. 5:**
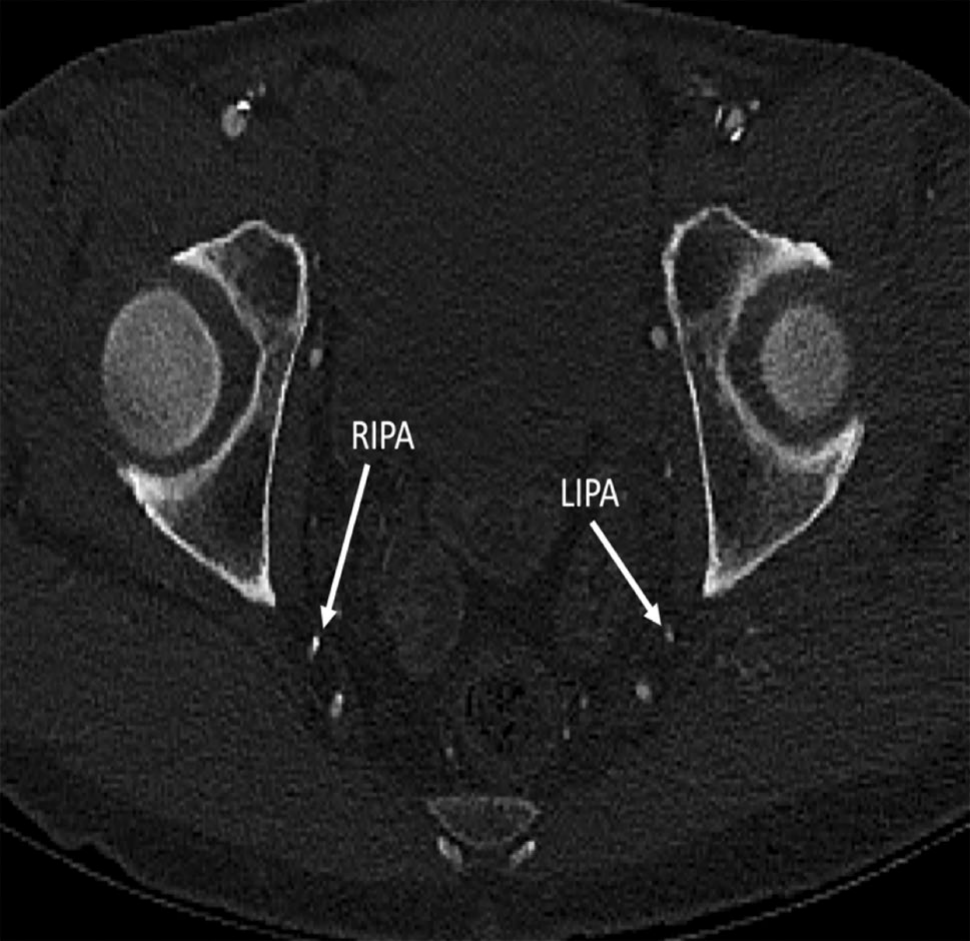
Post-contrast CT images of a 52-year-old male smoker, who scored 10/10 on calcium scoring. Heavy arterial vascular calcification was demonstrated within multiple vessels bilaterally, including the proximal and distal abdominal aorta, common iliac arteries, internal and external iliac arteries, and the internal pudendal arteries. RIPA/LIPA right internal pudendal artery/left internal pudendal artery

The association between vascular calcification, specifically coronary artery, or peripheral arterial calcification, and smoking is well researched, as is radiological imaging of same [15, 16]. Tobacco companies have also acknowledged these potential associations and risks. The link between IPA calcification and ED is also very well studied [1]. However, based on our literature review, there is a dearth of research available outlining any direct relationship between smoking and IPA calcification. Given the strong association between smoking, and IPA calcification in our study group, we further hypothesise a potential further association between smoking and ED, which has not been previously discussed, studied, or acknowledged by the tobacco industry (Fig. 5).

It is well established that the most common factors causing vascular calcification include (but are not limited to) diabetes, renal impairment, smoking, hypertension, hyperlipidaemia, and increased age [17]. In order to reduce confounding factors and biases as much as feasibly possible, we excluded all diabetics and all patients with any form of renal impairment from this study, which may have skewed the results. We also excluded any ex-smokers, as data was not available on when patients gave up smoking, or how much they had smoked previously. Also, our aim was to specifically strictly compare non-smokers, versus smokers. The age range of 40–60 years of age was also specifically chosen, as those under 40 are unlikely to have minimal if any vascular calcification, regardless of smoking history. Similarly, those over the age of 60 can demonstrate some mild physiological age-related vascular calcification, again regardless of whether they smoked or not. We hoped that targeting the 40–60 year age group would provide a more true reflection of the specific effect of smoking, and to minimise age related changes. History of hypertension or hyperlipidaemia was not evaluated however which was a limitation of this study. Another limitation was the quantity of smoking for the smoking group; we did not quantify the smoking “pack year history” for each patient as this data was not available. It would be beneficial to compare the degree of vascular calcification, compared with the amount of pack year smoking history, which we endeavour to evaluate in further research moving forward. Also, the calcium scores were assigned by only one radiologist. A double or triple assessment would have been more advantageous.

Taking the limitations into account, the results from this novel study demonstrate a strong and statistically significant increase in the rate of IPA calcification in smokers, as compared to non-smokers, with an inferred indirect association with ED. Young to middle aged men should be made more aware of this potential risk, and we feel that this potential side effect should be as well publicised as heart disease and stroke currently is. These findings represent a unique and useful deterrent for health authorities to include in anti-smoking campaigns, to better inform the public, and hopefully reduce the incidence of smoking, particularly in men. Future studies in this area are needed to potentially image men with known ED, to assess their burden of vascular calcification.
